# Classifying home care clients’ risk of unplanned hospitalization with the resident assessment instrument

**DOI:** 10.1007/s41999-022-00665-x

**Published:** 2022-06-27

**Authors:** Jukka K. Rönneikkö, Heini Huhtala, Harriet Finne-Soveri, Jaakko N. Valvanne, Esa R. Jämsen

**Affiliations:** 1 Ylöjärvi Health Care Centre, Ylöjärvi Health Centre, Mikkolantie 10, 33470 Ylöjärvi, Finland; 2 Faculty of Social Sciences, Tampere University, Tampere, Finland; 3 National Institute for Health and Welfare, Helsinki, Finland; 4 Faculty of Medicine and Health Technology and Gerontology Research Center (GEREC), Tampere University, Tampere, Finland; 5 Department of Geriatrics, Tampere University Hospital, Tampere, Finland

**Keywords:** Hospitalization, RAI assessment, Home care, Case-finding tool, DIVERT

## Abstract

**Purpose:**

To identify predictive case finding tools for classifying the risk of unplanned hospitalization among home care clients utilizing the Resident Assessment Instrument-Home Care (RAI-HC), with special interest in the Detection of Indicators and Vulnerabilities for Emergency Room Trips (DIVERT) Scale.

**Methods:**

A register-based, retrospective study based on the RAI-HC assessments of 3,091 home care clients (mean age 80.9 years) in the City of Tampere, Finland, linked with hospital discharge records. The outcome was an unplanned hospitalization within 180 days after RAI-HC assessment. The Area Under the Curve (AUC) and the sensitivity and specificity were determined for the RAI-HC scales: DIVERT, Activities of Daily Living Hierarchy (ADLh), Cognitive Performance Scale (CPS), Changes in Health, End-Stage Diseases, Signs, and Symptoms Scale (CHESS), and Method for Assigning Priority Levels (MAPLe).

**Results:**

Altogether 3091 home care clients had a total of 7744 RAI-HC assessments, of which 1658 (21.4%) were followed by an unplanned hospitalization. The DIVERT Scale had an AUC of 0.62 (95% confidence interval 0.61–0.64) when all assessments were taken into account, but its value was poorer in the older age groups (< 70 years: 0.71 (0.65–0.77), 70–79 years: 0.66 (0.62–0.69), 80–89 years: 0.60 (0.58–0.62), ≥ 90 years: 0.59 (0.56–0.63)). AUCs for the other scales were poorer than those of DIVERT, with CHESS nearest to DIVERT. Time to hospitalization after assessment was shorter in higher DIVERT classes.

**Conclusion:**

The DIVERT Scale offers an approach to predicting unplanned hospitalization, especially among younger home care clients. Clients scoring high in the DIVERT algorithm were at the greatest risk of unplanned hospitalization and more likely to experience the outcome earlier than others.

**Supplementary Information:**

The online version contains supplementary material available at 10.1007/s41999-022-00665-x.

## Key summary points

**Aim:**

The aim was to identify tools for classifying the risk of unplanned hospitalization among home care clients utilizing the Resident Assessment Instrument-Home Care (RAI-HC).

**Findings:**

The Detection of Indicators and Vulnerabilities for Emergency Room Trips (DIVERT) Scale predicts unplanned hospitalizations in home care clients. In the oldest age groups, however, it works poorly.

**Message:**

DIVERT Scale can be used for identifying high-risk home care clients needing urgent care planning to prevent unplanned hospital admissions and their potential adverse consequences. Clients scoring high in the scale and experiencing the outcome earlier than others, should be the primary group for more detailed assessment.

**Supplementary Information:**

The online version contains supplementary material available at 10.1007/s41999-022-00665-x.

## Introduction

Unplanned hospitalizations and emergency room visits are common among home care clients and are often associated with adverse outcomes [1]. In this population, the rate of hospitalization ranges from 17 to 38% in a follow-up of 2–6 months [2, 3] to 43% in a follow-up of one year [4]. Although hospitalizations are often due to acute exacerbations of chronic diseases [5], an earlier study among new home care clients indicated that several well-known geriatric challenges also predict unplanned hospitalization [4]. Identifying and managing modifiable conditions could provide a means to prevent unplanned hospital admissions [6].

A prognostic tool for identifying home care clients at high risk of unplanned hospitalization could help targeting comprehensive assessment to those in the most urgent need. However, to the best of authors’ knowledge, none of the previously described prognostic case-finding scales [7–15] have been validated for the frail population needing home care services.

Because emergency department (ED) visits of old patients often lead to hospitalization [16, 17] and the risk factors for ED visits and hospitalization are partly the same [4, 18], a scale predicting ED use could also identify clients at risk for unplanned hospitalization. The Detection of Indicators and Vulnerabilities for Emergency Room Trips (DIVERT) Scale, based on the Resident Assessment Instrument for Home Care (RAI-HC), is a valid case-finding algorithm for ED use in older home care clients [18]. This study aimed to determine the accuracy of DIVERT in predicting home care clients’ unplanned hospitalizations and to compare it to four validated RAI-HC scales in terms of their ability to classify the risk for hospitalization.

## Materials and methods

The Resident Assessment Instrument for Home Care (RAI-HC) is a comprehensive assessment instrument, developed to identify the needs of home care clients with disabilities. The RAI-HC collects information on the service use of clients and the clients’ physical, mental, social, and cognitive domains of health [19]. Its reliability and validity have been tested in international studies [19–21]. In earlier studies, some scales of the RAI-HC instrument have been associated with negative outcomes or the risk for unplanned hospitalization among home care clients [4, 22–25].

This study was based on the RAI-HC index assessments (*n* = 7744) made for home care clients (*n* = 3091) in the city of Tampere, Finland (ca. 240,000 inhabitants, of which 17% are aged 65 years or older) between January 1, 2014 and December 31, 2015. According to Finnish national guidelines, trained nurses perform the assessments at admission and then every six months or when there is a significant change in the client’s health status. The competence of the nurses carrying out the assessments will be ensured and the training of a new employee includes the RAI online course, exam, and the exercise assessment. After that, he/she performs the first client assessment together with an experienced nurse and learns about the results and how to utilize them.

Data about hospitalizations occurring within 180 days after RAI-HC assessment were collected from the mandatory hospital discharge records of Tampere University Hospital and the secondary and primary care wards of the City of Tampere, and they were linked to the RAI-HC data using each patient’s unique identification number. The hospitals represent public health care and cover all unplanned inpatient care within the area, regardless of social or insurance status.

The six-level DIVERT Scale has been developed for classifying the risk of ED admission in older home care clients. The Scale is based on an algorithm generated from RAI-HC data and includes previous ED use, cardiorespiratory symptoms, cardiac conditions, diagnoses of stroke, diabetes, renal failure, pneumonia, chronic obstructive pulmonary disease, and urinary tract infection and certain geriatric symptoms and syndromes: mood symptoms, falls, poor nutrition, skin ulcers, and ADL decline [18]. Like the standard RAI-HC scales, higher scores indicate a worse condition.

In addition to DIVERT, four RAI-HC scales previously associated with negative health outcomes among home care clients [4, 22–25] were used in this study and were compared to the DIVERT: activity of daily living performance (Activities of Daily Living Hierarchy (ADLh)) [26], cognitive performance (Cognitive Performance Scale (CPS)) [27], decision-support system for allocating home care resources (Method for Assigning Priority Levels (MAPLe)) [25], and health stability (Changes in Health, End-Stage Disease, Signs, and Symptoms (CHESS) Scale) [28].

The primary outcome was an unplanned hospitalization within the 180 days after the RAI-HC assessment. If a client met the outcome, he/she was excluded from further follow-up and later RAI-HC assessments were ignored. Data formation is described in Online Resource 1. Scheduled hospitalizations (e.g., elective surgery) were not taken into account, because the aim was to analyze only unplanned hospitalizations.

RAI data has been collected to a national register since 2000, held by the Finnish Institute of Health and Welfare, right under Ministry of Social Affairs and Health. At the time of founding the register, an ethical approval to collect these data twice every year until 2023, was obtained from the Ministry of Social Affairs and Health. From 2023 on, collection of RAI-data will be legislation based, and mandatory, in the country. The use of the RAI database and hospital discharge data in this study was approved by the authorities of City of Tampere (decisions of Director of Hospital Services December 16, 2014, August 30, 2016 and June 16, 2017, and Director of Services for the Aged June 20, 2017), and Tampere University Hospital (R20613). Because of the retrospective, register-based nature of this study, ethics board approval or home care clients’ informed consent was not required, according to national and European Union legislation.

### Statistical analyses

The association between the DIVERT Scale and hospitalization was first investigated with logistic regression. To compare the predictive accuracy of DIVERT and the RAI-HC scales in relation to the study outcome, Receiver Operating Character Curves (ROC) were then calculated, of which the areas under the receiver operating characteristic curve (AUC) are presented for all analyzed scales. The analyses were done for the whole data and separately for different age groups (< 70, 70–79, 80–89, ≥ 90 years). Finally, median time from assessment to hospitalization was determined and compared across three risk levels: low risk of hospitalization (DIVERT levels 1–2), moderate risk (DIVERT 3–4) and high risk. The statistical analyses were performed using SPSS version 25 (IBM Corp, Armonk, NY).

## Results

Of the 7744 RAI-HC assessments (for 3091 home care clients), 1658 (21%) were followed by an unplanned hospitalization within 180 days after the assessment, and altogether 54% of the clients were hospitalized at least once during the study period up. Of the assessments, 1,045 (14%) were in the age group < 70 years, 1658 (21%) in 70–79 years, 3,857 (50%) in 80–89 years and 1184 (15%) in ≥ 90 years. Of the 1,658 clients hospitalized, 81 (5%) were < 70 years, 286 (17%) 70–79 years, 915 (55%) 80–89 years, and 376 (23%) ≥ 90 years. Table 1 shows the characteristics of the home care clients at the time of their first RAI-HC assessment during the study period.

**Table 1:** Characteristics of the assessed home care clients based on their first RAI-HC assessment of the study period

	ALL N	%
	3,091	100
*Demographics*		
Mean age (years)	80.9 SD 9.9	
*Age*		
< 70	428	13.8
70–79	691	22.4
80–89	1,532	49.6
90 + ^b^	440	14.2
*Sex*		
Female	2,144	69.4
Male^b^	947	30.6
*Social situation*		
Housing-related problems^b^	92	3.0
Caregiver stressed	139	4.5
*Use or needs of services*		
Acute outpatient care or unplanned hospitalization in 90 days before assessment^a b^	1,546	50.0
*Method for assigning priority levels score*		
1–2	975	31.5
3	514	16.6
4	1,135	36.7
5	467	15.1
*Function*		0.0
ADL decline in previous 90 days^a^	1,003	32.4
*Activities of daily living hierarchy score*		
0	2,510	81.2
1–2	353	11.4
3–4	187	6.0
5–6	41	1.3
*Poor prospects for functional improvement*^a^	2,828	91.5
Poor self-reported health ^b^	841	27.2
*Cognitive performance scale score*		
0	1,019	33.0
1–2^b^	1,774	57.4
3–4^b^	209	6.8
5–6^b^	89	2.9
*Clinical symptoms*		
Any cardio-respiratory symptoms^a^	1,089	35.2
Urinary incontinence daily^b^	646	20.9
Urinary catheter^a^	1	0.03
Fecal incontinency^b^	181	5.9
Stasis ulcers^a b^	150	4.9
Falls during 90 days before assessment^a b^	777	25.1
Any mood symptoms^a^	1,305	42.2
*Pain Scale score*		
0–1	1,944	62.9
2–3^b^	1,147	37.1
Weight loss^a^	138	4.5
Decrease in food or fluids^a^	119	3.8
*Body mass index, kg/m*^*2*^		
< 18.5	145	4.7
18.5–23.9	905	29.3
24–29.9	1,187	38.4
≥ 30	723	23.4
*Special therapies*		
Oxygen therapy^a^	25	0.8
Diagnoses		
Congestive heart failure^a b^	655	21.2
Coronary artery disease^a b^	723	23.4
Alzheimer's disease	794	25.7
Other dementia	320	10.4
History of stroke^a^	207	6.7
Parkinson's disease^b^	73	2.4
Musculoskeletal disorders	1,005	32.5
Cancer^b^	261	8.4
Renal insufficiency^a,b^	268	8.7
Psychiatric diagnosis	636	20.6
Chronic obstructive pulmonary disease^a,b^	363	11.7
Diabetes^a^	942	30.5
Pneumonia^a^	75	2.4
History of urinary tract infection^a^	21	0.7
*Medication*		
*Number of drugs*^c^		
0–4	333	10.8
5–8^b^	1,013	32.8
9 or more^b^	1,745	56.5
Psychotropic medication	1,720	55.6
*Health stability*		
*Changes in Health, End-Stage Disease, Signs, and Symptoms Scale score*		
0	1,294	41.9
1^b^	861	27.9
2–5^b^	936	30.3

^a^Variables included in the DIVERT algorithm

^b^Independent risk factors for hospitalization in a previous study (4)

^c^Including prescription and non-prescription medications

As indicated in Table 2, clients with high DIVERT scores were at the greatest risk of hospitalization. Although the absolute risk of hospitalization increased with age, the association with the DIVERT levels was lower in the higher age groups (Online Resource 2).

**Table 2:** Distribution of DIVERT scores and absolute risk, sensitivity, specificity and odds ratio of unplanned hospitalization, according to DIVERT score

DIVERT Level	Number of assessments		Number of outcomes		Sensitivity	Specificity	OR	95% CI
	N	%	N	%				
1	1,591	20.5	174	10.9			1	
2	1,992	25.7	364	18.3	0.90	0.23	1.82	1.50–2.21
3	1,437	18.6	298	20.7	0.68	0.50	2.13	1.74–2.61
4	1,166	15.1	320	27.4	0.50	0.69	3.08	2.51–3.78
5	894	11.5	258	28.9	0.30	0.83	3.30	2.67–4.09
6	664	8.6	244	36.7	0.15	0.93	4.73	3.79–5.91
Total	7,744	100.0	1,658	21.4				

**Fig. 1:**
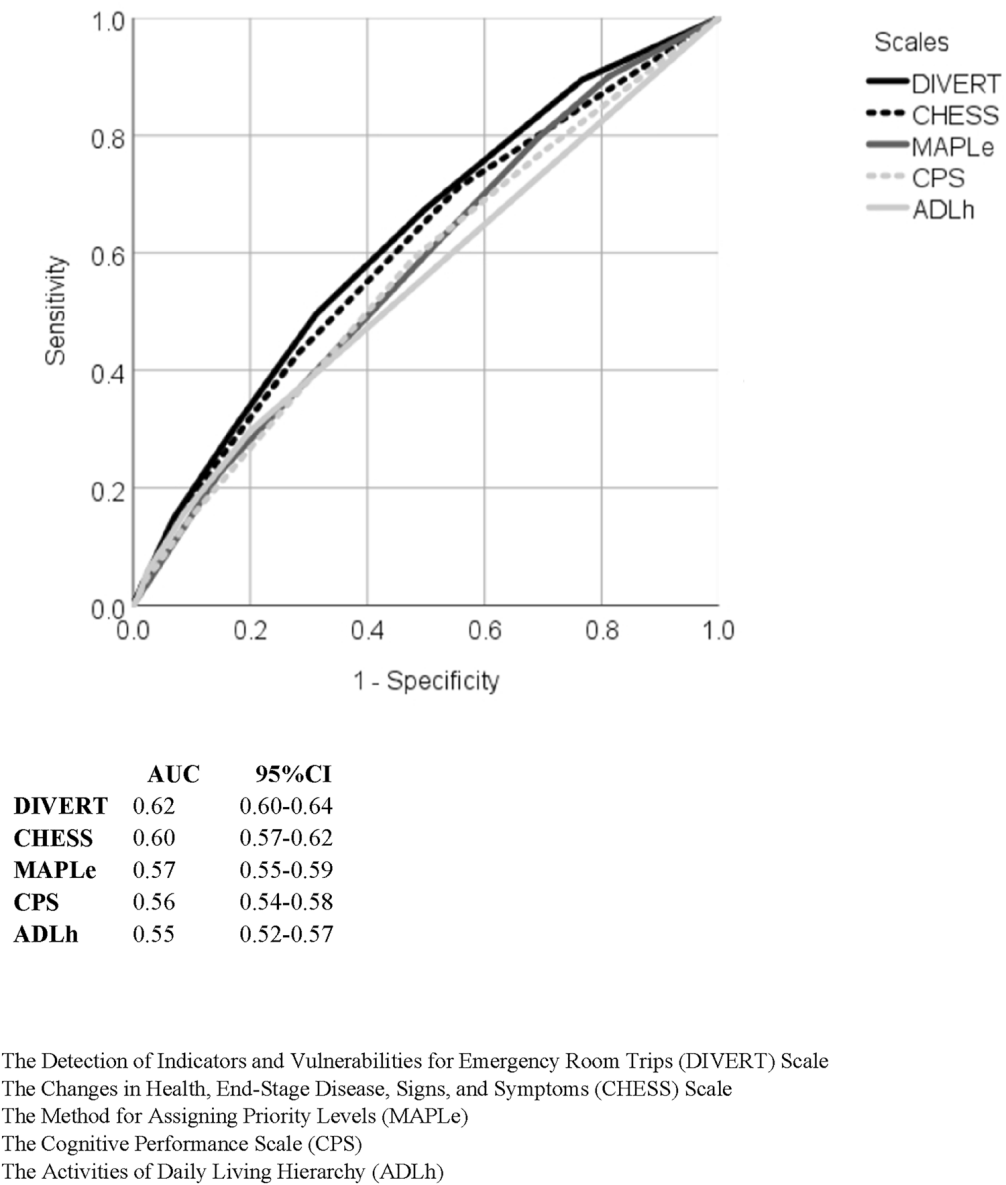
ROC curves and values of AUC for all scales in whole data

**Fig. 2:**
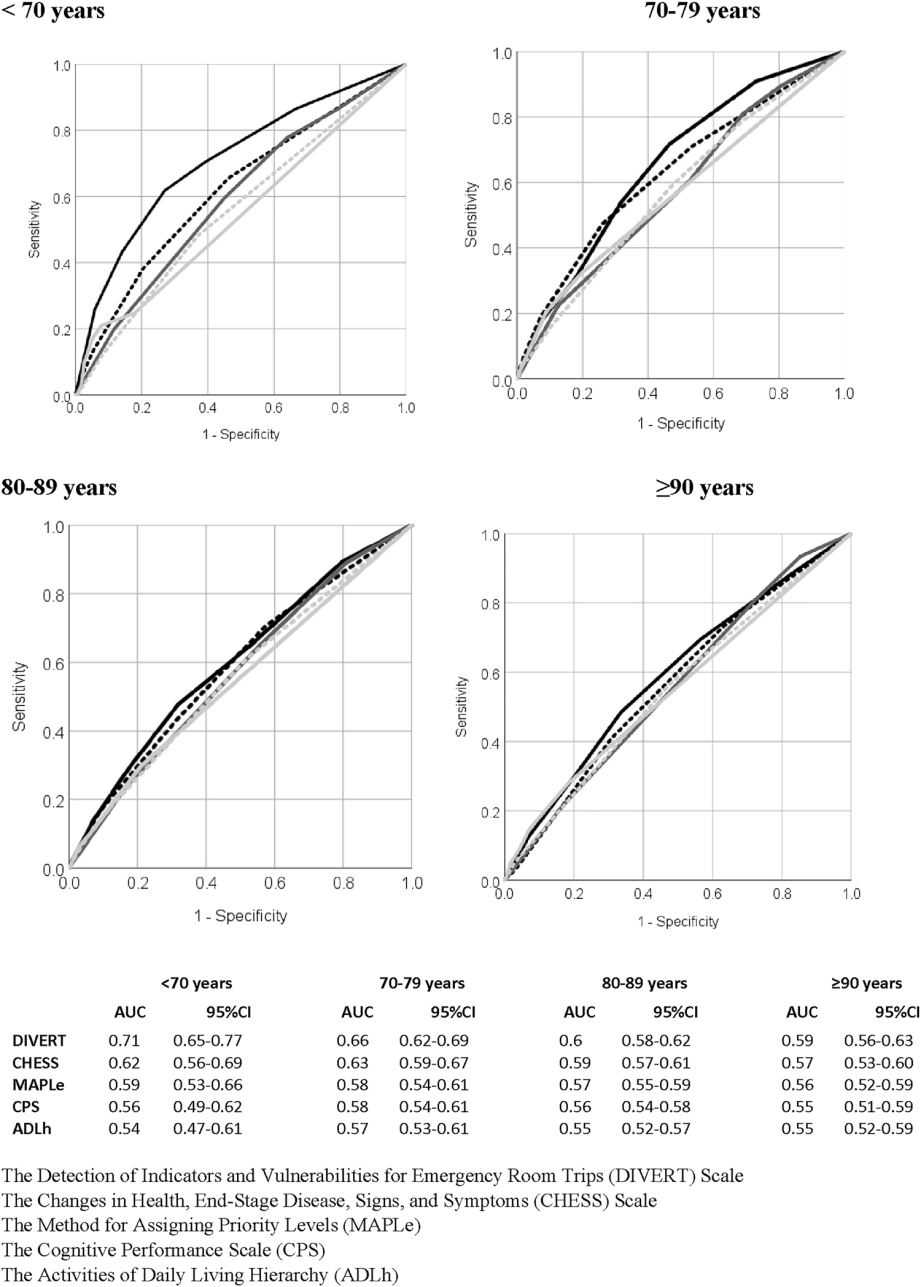
ROC curves and values of AUC for DIVERT (solid black line), CHESS (dashed black line), MAPLe (solid dark gray line), CPS (dashed gray line) and ADLh (solid gray line) in different age groups

In the whole data, the DIVERT Scale had an AUC of 0.62 (95% confidence interval 0.60–0.64) (Fig. 1). The predictive accuracy was better in clients aged < 70 years (0.71 (0.65–0.77)) than in the older age groups (70–79 years: 0.66 (0.62–0.69), 80–89: years 0.60 (0.58–0.62), ≥ 90 years: 0.59 (0.56–0.63)) (Fig. 2).

The AUCs for the ADLh, CPS, and MAPLe scales ranged from 0.55 to 0.58 (Fig. 1). CHESS was closest to DIVERT (0.60 (0.57–0.62)). None of the scales had better predictive validity than DIVERT in the any of the analyzed age groups (Fig. 2).

Among the hospitalized clients, the median times from assessment to hospitalization were 45 days, 66 days and 72 days for those with high (DIVERT 5–6; *n* = 502), moderate (DIVERT 3–4; *n* = 618) and low (DIVERT 1–2; *n* = 538) risk, respectively (*p* < 0.001).

## Discussion

This study confirmed that the DIVERT Scale, a case-finding algorithm primarily validated for ED use, has the same relatively low predictive accuracy (AUC 0.62) in differentiating the risk of unplanned hospitalization as in a previous study concerning ED use [18]. However, the accuracy was better in clients aged < 70 years (AUC 0.71). Clients with high DIVERT scores were at the greatest risk and also experienced the outcome earlier than others.

Screening instruments for identifying home-dwelling old people at risk of hospitalization have been developed and validated in previous studies. These instruments are based on self-reported information about medical conditions [8, 10–12], electronic medical records [7, 14] and risk assessments made by a general practitioner [9]. The reported AUCs have ranged from 0.62 to 0.74 (poor or moderate accuracy) depending on the assessment tool, population, setting and follow-up. The AUCs of DIVERT in this study are hence at the lower (i.e., poorer) end of the previously reported range. This may be at least partly explained due to the different target population. In contrast to previous studies, all patients in our study received home care services and often had previous acute outpatient care or hospitalizations, indicating more unstable health condition.

The reasons for the poorer accuracy of DIVERT in the older age groups are partly obscure.

It is possible that the major geriatric challenges, such as frailty and cognitive impairment, not included in DIVERT are both common and have a dominant role in explaining the risk of hospitalization in the higher age groups, whereas DIVERT emphasizes cardiovascular diseases and other disease-related factors that may be more important in the younger age groups. For example, frailty has been linked to an increased risk for multiple adverse health-related outcomes, including hospital admissions [29]. However, the potential utility of frailty scales in identifying the risk for hospitalization is unclear [23]*.* Information about how conditions not included in the current algorithm affect the risk of hospitalization in older clients at different DIVERT levels could help improving the accuracy of the algorithm. Moreover, such information could also reveal possible targets for interventions to reduce the risk of ED and hospital admission.

Despite its limitations, DIVERT performed better than the analyzed RAI-HC scales that have previously been associated with negative outcomes among home care clients [4, 22–25]. Although the CHESS score, a measure of health stability and an indicator of functional decline [29], was an independent risk factor for hospitalization in an earlier study [4], the present study indicated low accuracy in the prediction of hospitalization, supporting previous observations [23, 30]. CPS, ADLh and MAPLe scales had even lower accuracy and they are not useful in the risk assessment alone as such.

In spite of the relatively low positive predictive power of available screening tools, case management programs based on these instruments can be cost-effective, depending on the costs of the programs and the anticipated savings [31]. Considering the high direct costs of hospital care and associated adverse outcomes (32), the DIVERT Scale could be used as a screening tool for the risk of hospitalization in the hope of the net savings that the case management will generate. Because the absolute risk of hospitalization of clients with a lower DIVERT score was small, it would be sensible to plan and target predictive strategies to clients with DIVERT scores 5–6. As those clients were also hospitalized in a shorter time than those at low risk (DIVERT 1–2), they should be the primary group for a more detailed assessment.

This research is based only on RAI-HC data from a single city, limiting its generalizability to rural areas and other countries. On the other hand, the data have good coverage: only circa 15% of home care clients in the catchment area were not included due to a missing RAI-HC assessment. The data also represents well typical home care clients in an urban area. The types and availability of services were the same in the whole area, so they do not affect hospital utilization rates, and thanks to public health insurance, the clients’ economic and social status do not affect their access to public health care either. According to national instructions, a new RAI-HC assessment should be performed when there is a significant change in the client’s health status. The number of included assessments suggest that RAI-HC assessments were not fully made in accordance with these guidelines. If a client’s health status had changed after the assessment, the assessment may not have reflected the client’s real condition, possibly leading to misclassification on the DIVERT and RAI-HC scales.

## Conclusion

The DIVERT Scale has the same, somewhat limited predictive accuracy in differentiating the risk of unplanned hospitalization as in a previous study concerning ED admissions. However, it could be used for identifying high-risk clients needing urgent care planning to prevent hospital admissions and their potential adverse consequences in this vulnerable population. In older age groups, the value of the DIVERT Scale is poorer, possibly because it does not take geriatric syndromes and frailty into account.

## Supplementary Information

Below is the link to the electronic supplementary material.

Supplementary file1 (DOCX 32 KB)

Supplementary file2 (DOCX 26 KB)
