# Supplementary material for: Classifying home care clients’ risk of unplanned hospitalization with the resident assessment instrument
Source: Eur Geriatr Med. 2022 Jun 27;13(5):1129–36. doi: 10.1007/s41999-022-00665-x (PMC9553799; doi:10.1007/s41999-022-00665-x)
Supplement: Supplementary file 1 — Supplementary file1 (DOCX 32 KB) [file 41999_2022_665_MOESM1_ESM.docx]

| **ESM_1. The sequence number of the RAI-HC assessments and the number of clients assessed and hospitalized respectively.**  If a client met the outcome, he/she was excluded from further follow-up and later RAI-HC assessments were ignored. | | | | |  |
| --- | --- | --- | --- | --- | --- |
|  |  |  |  |  |  |
|  |  |  |  |  |  |
|  |  |  |  |  |  |
|  |  |  |  |  |  |
| **The sequence number of the RAI-HC assessment** |  | **n=The number of clients assessed** |  | **n=The number of clients hospitalized in the 180 days after assessment** |  |
|  |  |  |  |  |  |
|  |  |  |  |  |  |
|  |  |  |  |  |  |
| 1st |  | n=3,091 | \|  \| \| --- \| | n=704 |  |
|  |  |  |  |  |  |
| \|  \| \| --- \| |  | \|  \| \| --- \| |  |  |  |
|  |  |  |  |  |  |
| 2nd |  | n=2,180 | \|  \| \| --- \| | n=481 |  |
|  |  |  |  |  |  |
| \|  \| \| --- \| |  | \|  \| \| --- \| |  |  |  |
|  |  |  |  |  |  |
| 3rd |  | n=1,532 | \|  \| \| --- \| | n=327 |  |
|  |  |  |  |  |  |
|  |  |  |  |  |  |
|  |  |  |  |  |  |
| 4th |  | n=777 | \|  \| \| --- \| | n=123 |  |
|  |  |  |  |  |  |
| \|  \| \| --- \| |  | \|  \| \| --- \| |  |  |  |
|  |  |  |  |  |  |
| 5th |  | n=150 | \|  \| \| --- \| | n=22 |  |
|  |  |  |  |  |  |
| \|  \| \| --- \| |  | \|  \| \| --- \| |  |  |  |
|  |  |  |  |  |  |
| 6th |  | n=12 | \|  \| \| --- \| | n=1 |  |
|  |  |  |  |  |  |
| \|  \| \| --- \| |  | \|  \| \| --- \| |  |  |  |
|  |  |  |  |  |  |
| 7th |  | n=2 | \|  \| \| --- \| | n=0 |  |
|  |  |  |  |  |  |
