# Supplementary material for: Classifying home care clients’ risk of unplanned hospitalization with the resident assessment instrument
Source: Eur Geriatr Med. 2022 Jun 27;13(5):1129–36. doi: 10.1007/s41999-022-00665-x (PMC9553799; doi:10.1007/s41999-022-00665-x)
Supplement: Supplementary file 2 — Supplementary file2 (DOCX 26 KB) [file 41999_2022_665_MOESM2_ESM.docx]

| \| **ESM_2. Distribution of DIVERT scores and absolute risk, sensitivity, specificity and odds ratio of unplanned hospitalization, according to DIVERT score in different age groups** \| \| --- \| \| \| \|  \| \| <70 years \|  \| | | | | | | | | | |
| --- | --- | --- | --- | --- | --- | --- | --- | --- | --- | --- | --- | --- | --- |
| **DIVERT Level** | **Number of assessments** | |  | **Number of outcomes** | | **Sensitivity** | **Specificity** |  |  |
|  |  |  |  |  |  |  |  | **OR** | **95% CI** |
|  | **N** | **%** |  | **N** | **%** |  |  |  |  |
| **1** | 334 | 32.0 |  | 11 | 3.3 |  |  | 1 |  |
| **2** | 276 | 26.4 |  | 13 | 4.7 | 0.86 | 0.34 | 1.45 | 0.64-3.29 |
| **3** | 125 | 12.0 |  | 7 | 5.6 | 0.70 | 0.61 | 1.74 | 0.66-4.60 |
| **4** | 139 | 13.3 |  | 15 | 10.8 | 0.62 | 0.73 | 3.55 | 1.59-7.95 |
| **5** | 93 | 8.9 |  | 14 | 15.1 | 0.43 | 0.86 | 5.20 | 2.28-11.90 |
| **6** | 78 | 7.5 |  | 21 | 26.9 | 0.26 | 0.94 | 10.82 | 4.95-23.65 |
| **Total** | 1,045 | 100.0 |  | 81 | 7.8 |  |  |  |  |

| \|  \| \| --- \| \|  \| \| 70-79 years \|  \| | | | | | | | | | |
| --- | --- | --- | --- | --- | --- | --- | --- | --- | --- | --- | --- | --- | --- |
| **DIVERT Level** | **Number of assessments** | |  | **Number of outcomes** | | **Sensitivity** | **Specificity** |  |  |
|  |  |  |  |  |  |  |  | **OR** | **95% CI** |
|  | **N** | **%** |  | **N** | **%** |  |  |  |  |
| **1** | 396 | 23.9 |  | 26 | 6.6 |  |  | 1 |  |
| **2** | 418 | 25.2 |  | 55 | 13.2 | 0.91 | 0.27 | 2.16 | 1.32-3.51 |
| **3** | 255 | 15.4 |  | 51 | 20.0 | 0.72 | 0.53 | 3.56 | 2.15-5.88 |
| **4** | 260 | 15.7 |  | 67 | 25.8 | 0.54 | 0.68 | 4.94 | 3.04-8.03 |
| **5** | 175 | 10.6 |  | 36 | 20.6 | 0.30 | 0.82 | 3.69 | 2.15-6.30 |
| **6** | 154 | 9.3 |  | 51 | 33.1 | 0.18 | 0.93 | 7.05 | 4.19-11.86 |
| **Total** | 1,658 | 100.0 |  | 286 | 17.2 |  |  |  |  |

| \|  \| \| --- \| \|  \| \| 80-89 years \|  \| | | | | | | | | | |
| --- | --- | --- | --- | --- | --- | --- | --- | --- | --- | --- | --- | --- | --- |
| **DIVERT Level** | **Number of assessments** | |  | **Number of outcomes** | | **Sensitivity** | **Specificity** |  |  |
|  |  |  |  |  |  |  |  | **OR** | **95% CI** |
|  | **N** | **%** |  | **N** | **%** |  |  |  |  |
| **1** | 697 | 18.1 |  | 98 | 14.1 |  |  | 1 |  |
| **2** | 995 | 25.8 |  | 220 | 22.1 | 0.89 | 0.20 | 1.74 | 1.34-2.25 |
| **3** | 793 | 20.6 |  | 161 | 20.3 | 0.65 | 0.47 | 1.56 | 1.18-2.05 |
| **4** | 593 | 15.4 |  | 170 | 28.7 | 0.48 | 0.68 | 2.46 | 1.86-3.24 |
| **5** | 452 | 11.7 |  | 142 | 31.4 | 0.29 | 0.83 | 2.80 | 2.09-3.75 |
| **6** | 327 | 8.5 |  | 124 | 37.9 | 0.14 | 0.93 | 3.73 | 2.74-5.09 |
| **Total** | 3,857 | 100.0 |  | 915 | 23.7 |  |  |  |  |

| \|  \| \| --- \| \|  \| \| **≥90 years** \|  \| | | | | | | | | | |
| --- | --- | --- | --- | --- | --- | --- | --- | --- | --- | --- | --- | --- | --- |
| **DIVERT Level** | **Number of assessments** | |  | **Number of outcomes** | | **Sensitivity** | **Specificity** |  |  |
|  |  |  |  |  |  |  |  | **OR** | **95% CI** |
|  | **N** | **%** |  | **N** | **%** |  |  |  |  |
| **1** | 164 | 13.9 |  | 39 | 23.8 |  |  | 1 |  |
| **2** | 303 | 25.6 |  | 76 | 25.1 | 0.90 | 0.16 | 1.07 | 0.69-1.67 |
| **3** | 264 | 22.3 |  | 79 | 29.9 | 0.69 | 0.44 | 1.37 | 0.88-2.14 |
| **4** | 174 | 14.7 |  | 68 | 39.1 | 0.48 | 0.67 | 2.06 | 1.28-3.29 |
| **5** | 174 | 14.7 |  | 66 | 37.9 | 0.30 | 0.80 | 1.96 | 1.22-3.14 |
| **6** | 105 | 8.9 |  | 48 | 45.7 | 0.13 | 0.93 | 2.70 | 1.60-4.57 |
| **Total** | 1,184 | 100.0 |  | 376 | 31.8 |  |  |  |  |
